# Improvement of lipid stability of refrigerated rainbow trout (*Oncorhynchus* *mykiss*) fillets by pre-storage α-tocopherol acetate dipping treatment

**Published:** 2012

**Authors:** Ali Ehsani, Mohammad Sedigh Jasour

**Affiliations:** 1 *Department of Food Hygiene, Faculty of Veterinary Medicine, and Department of Biotechnology and Quality Control, Artemia and Aquatic Animals Research Institute, Urmia University, Urmia, Iran;*; 2 *Department of Biotechnology and Quality Control, Artemia and Aquatic Animals Research Institute, Urmia University, Urmia, Iran.*

**Keywords:** Lipid stability, Dip treatments, Alpha-tocopherol acetate, Trout fillet, Food storage

## Abstract

One of the simplest methods for short-term handling and storage of fish is the refrigeration in combination with dip treatments. This study was conducted to determine the effect of pre-storage α-tocopherol acetate dipping treatments on lipid stability of refrigerated rainbow trout fillets. Trout fillets were dipped in α-tocopherol acetate solutions (200 and 500 mg kg^-1^ flesh) and subsequently stored in a refrigerator at 4 ˚C for 12 days. Control samples received no α-tocopherol acetate during dip treatment. At the end of 0, 3, 6, 9 and 12 days of storage, lipid damage analysis of trout fillets was performed. A continuous notable increase (*p* < 0.05) in peroxide value (PV), thiobarbituric acid (TBA) and free fatty acid (FFA) was observed for all samples throughout the storage period. Although throughout the storage period the lipid hydrolysis (FFA) of fish fillets was not affected by α-tocopherol acetate solutions, successful (*p* < 0.05) inhibition of lipid oxidation (PV and TBA) in refrigerated trout fillets was possible with dip treatment in α-tocopherol acetate solutions (200 and 500 mg kg^-1^ flesh). There was no significant (*p* > 0.05) effect of increasing α-tocopherol acetate concentration on the lipid deterioration of fish fillets. These results indicated that α-tocopherol acetate especially at lower level (200 mg kg^-1^ flesh) was very effective in retarding the lipid oxidation.

## Introduction

Consumers are demanding ever healthier and safer foods. Fish have beneficial effects on coronary heart disease, inflammatory and auto-immune disorders, brain development and mental health due to presence of high amount of poly unsaturated fatty acids (PUFA).^1^ On the other hand, PUFAs in fish fillet are very susceptible to rancidity caused by oxygen free radicals.^2^

Lipid oxidation is a major cause of quality deterioration in fish muscle during storage period.^3^ Frequently, fish and seafood products are stored for retail purposes under refrigeration conditions and the relatively short shelf life of fresh fish is the single greatest concern to retail fish markets.^4^ With increasing consumption of fish, control of oxidation has become increasingly important and efforts to find acceptable ways of limiting lipid oxidation are of great importance.^5^

Using of antioxidants is an effective way to minimize or delay the oxidation process, retarding the formation of toxic oxidation products, maintaining nutritional quality and prolonging the shelf-life of food.^6^ Alpha-tocopherol acetate is the synthetic form of vitamin E, inhibiting the lipid peroxidation by attaching free radicals to its double conjugated linkages.^7^ In the meat industry, dip treatments have been used to minimize microbial growth and retard color changes or lipid oxidation in the products prior to storage.^8^ It has been reported that dipping with α-tocopherol solutions improves lipid stability of beef and frozen mackerel fish.^9^^,^^10^ Also, addition of α- tocopherol acetate after slaughtering on fish fillet improved lipid stability of tilapia (*Oreochromis* *niloticus*) hamburgers during frozen storage.^11^

There is yet insufficient data on the direct treatment of freshwater fish with α-tocopherol acetate. Rainbow trout is the main aquacultured freshwater fish. This fish is highly demanded among the consumers in Iran. It is necessary to increase the shelf life and retain quality and nutritional attributes of economically important fish. Thus, this study was designed to determine the effect of pre-storage α-tocopherol acetate by dipping treatments on lipid stability of refrigerated rainbow trout fillets.

## Materials and Methods

**Sampling procedure and storage condition. **Sixty rainbow trout with mean initial weight of 255 ± 5 g were harvested in November 2010 from the aquaculture farm of Artemia and Aquatic Animal’s Research Institute (West Azerbaijan, Urmia-Iran). The fish were fed commercial diet containing 42.0% protein, 12.0% fat, 3.7% crude cellulose, 13.0% ash and 10.0% moisture (4 mm in diameter pellet, Fara-daneh Co., Esfahan-Iran). Fish were killed by ice shocking and less than 3 hours post-capture on arrival at the laboratory stored in ice. Fish were washed with tap water, descaled, beheaded and filleted and equally divided into three groups (20 in each): The first group defined as control group, that it is dipped in solution of ethanol and distilled water without any α-tocopherol acetate. Other two groups separately at the same time dipped in solutions of 200 and 500 mg of α-tocopherol acetate per kg of flesh for 5 min, and then drained for 60 sec. Preparation of α-tocopherol acetate solutions was performed by dissolving α-tocopherol acetate (Merck, Darmstadt, Germany) in ethanol (70% purity). The distilled water was used for dilution. Concentrations of α-tocopherol acetate were calculated by total flesh weight in each group. Two fillets were packed separately in polythene bags, and stored in a refrigerator at 4 ˚C for 12 days. For lipid extraction and lipid damage analysis, 8 randomly selected fillets from each group were homogenized in a kitchen meat mincer every 3 days, on day 0, 3, 6, 9 and 12 of refrigeration.

**Lipid extraction. **The total lipid was extracted from the fish muscle by the Bligh and Dyer method.^12^ A mixture of chloroform and methanol (2:1, v/v) was used as a solvent. 

**Lipid damage analysis. **The peroxide value (PV), thiobarbituric acid (TBA) and free fatty acid (FFA) content were determined in the extracted lipid. PV and FFA were determined according to the method of Egan *et al.* and expressed in miliequivalente (mEq) peroxide per kg of lipid and percent of oleic acid, respectively.^13^ According to the method of Kirk and Sawyer,^14^ TBA showed absorbance at 532 nm and results expressed as mg malonaldehyde (MDA) per kg of lipid.

**Statistical** **Analysis. **All experiments were run in triplicate and all data were presented as mean ± standard deviation (SD). The distribution of the data was performed by the Kolmogorov–Smirnov normality test and then subjected to one way analysis of variance (ANOVA). Duncan test was performed for differences between means. The differences between the means were considered statistically significant for *p*-values < 0.05. All statistical analysis was conducted using PASW Statistics version 18.0 for Windows (SPSS Inc., Chicago, IL, USA). 

## Results

In Table 1 the PV, TBA and FFA values of rainbow trout fillets subjected to dipping treatments during 12 days of refrigeration are presented. At time 0, no difference in lipid characteristics was observed between treatments and PV, TBA and FFA values of all samples ranged from 1.11 to 1.14 mEq kg^-1^, 0.05 to 0.06 mg MDA per kg and 1.69 to 1.80%, respectively. A continuous notable increase (*p* < 0.05) in PV, TBA and FFA was found for all samples throughout the storage period; PV, TBA and FFA values of all samples ranged from 15.06 to 22.08 mEq kg^-1^ lipid, 0.97 to 1.38 mg MDA per kg lipid and 7.01 to 7.08%, respectively Although throughout the storage period the FFA of fish fillets was not affected (*p* > 0.05) by α-tocopherol acetate solutions, the PV and TBA levels were found to be low (*p* < 0.05) in samples that dipped in α-tocopherol acetate solutions. The samples that received the high level of α-tocopherol acetate (500 mg kg^-1^) showed lower formation of PV, TBA and FFA, but there was no significant (*p* > 0.05) effect of increasing α-tocopherol acetate concentration on the lipid deterioration of fish fillets. These results indicated that α-tocopherol acetate especially at lower level (200 mg kg^-1 ^flesh) was very effective in retarding the lipid oxidation (Table 1).

**Table 1:** Changes in PV (mEq kg^-1 ^lipid), TBA (mg MDA per kg lipid) and FFA (% of oleic acid) values of rainbow trout fillets as affected by α-tocopherol acetate solutions (0, 200 and 500 mg kg^-1 ^flesh) during 12 days of refrigeration. Data are presented as mean ± SD (n = 3).

**Parameters**	**Treatments**	**Storage period (days)**				
		0	3	6	9	12
**PV**	0	1.14 ± 0.09^a^^E^	3.91 ± 0.06^a^^D^	9.30 ± 0.35^a^^C^	15.80 ± 0.07^a^^B^	22.08 ± 0.32^a^^A^
	200	1.13 ± 0.05^a^^E^	3.75 ± 0.05^b^^D^	7.80 ± 0.09^b^^C^	11.80 ± 0.11^b^^B^	15.30 ± 0.15^b^^A^
	500	1.11 ± 0.11^a^^E^	3.64 ± 0.12^b^^D^	7.78 ± 0.11^b^^C^	11.76 ± 0.09^b^^B^	15.06 ± 0.19^b^^A^
**TBA**	0	0.06 ± 0.04^a^^E^	0.37 ± 0.07^a^^D^	0.57 ± 0.08^a^^C^	0.81 ± 0.05^a^^B^	1.38 ± 0.05^a^^A^
	200	0.05 ± 0.07^a^^E^	0.19 ± 0.12^b^^D^	0.34 ± 0.06^b^^C^	0.65 ± 0.09^b^^B^	1.03 ± 0.09^b^^A^
	500	0.06 ± 0.04^a^^E^	0.17 ± 0.09^b^^D^	0.32 ± 0.07^b^^C^	0.63 ± 0.13^b^^B^	0.97 ± 0.11^b^^A^
**FFA**	0	1.80 ± 0.34^a^^E^	2.09 ± 0.74^a^^D^	3.58 ± 0.27^a^^C^	4.17 ± 0.31^a^^B^	7.08 ± 0.33^a^^A^
	200	1.72 ± 0.53^a^^E^	2.08 ± 0.48^a^^D^	3.45 ± 0.57^a^^C^	4.14 ± 0.23^a^^B^	7.04 ± 0.66^a^^A^
	500	1.69 ± 0.26^a^^E^	2.07 ± 0.36^a^^D^	3.44 ± 0.44^a^^C^	4.12 ± 0.45^a^^B^	7.01 ± 0.44^a^^A^

A-E Different letters within each storage time differs significantly (*p* < 0.05);

a-b Different letters within each treatment differs significantly (*p *< 0.05).

## Discussion

Lipid deterioration due to progressive oxidation and enzymatic hydrolysis, as the main cause of shortened shelf-life of fish and fish products are evaluated by means of the PV, TBA and FFA.^3^^,^^15^^,^^16^ Numerous factors such as the species, storage conditions (temperature and light) and fat composition can affect lipid oxidation in fish.^17^

The PV and TBA values measure primary and secondary products of lipid oxidation, respectively.^18^^,^^19^ The activity of an antioxidant can be estimated by quantitatively determining PV or TBA of lipids.^20^ The result of present study is in agreement with Helena and Silvia reported postmortem addition of α-tocopherol acetate (100 mg kg^-1^) improved lipid stability (i.e. TBA) of tilapia hamburgers during frozen storage.^11^ Also, Dragoev found lower contents of TBA in lipids of mackerel fish dipped with 200 mg α-tocopherol during frozen storage period.^10^ Furthermore, our results are in accordance with the results of Abou-Arab and Abu-Salem,^21^ who found that steaks from ostrich carcasses treated with 0.08% α-tocopherol acetate showed lower PV and TBA compared the control treatments. Tseng *et al.* also suggested that red claw crayfish tails dipped in α-tocopherol solutions (0.06% w/w) produced less TBA than aerobically packaged, water-dipped tails.^22^

The TBA value is generally regarded as a remarkable indicator for determining of deterioration of the organoleptic characteristics of meat.^23^ It has been reported that the maximum level of TBA value indicating good quality of the fish during storage period is 1-2 mg MDA per kg lipid.^24^ In the current study, final values of TBA reached to the level of tolerable maximum acceptable amounts at the end of storage period. 

Formation of free fatty acids (FFA); a result of enzymatic and non-enzymatic lipid hydrolysis is used as lipid quality indicator.^25^ FFA formation often occurs as a result of catalysis by endogenous enzymes.^26^ According to our observation even the low temperature (4 ˚C) in combination with α-tocopherol acetate solutions could not stop lipid hydrolysis. Published reports on the effect of α-tocopherol acetate on the FFA formation in fish are limited and this aspect needs further investigation. Despite the negative effect of FFA on protein solubility and texture deterioration, the formation of FFA itself does not lead to nutritional losses.^27^ In agreement with our results, progressive development of lipid hydrolysis was reported for rainbow trout (*Oncorhynchus* *mykiss*), Beluga sturgeon (*Huso* *huso*), carp (*Cyprinus* *carpio*) and mackerel (*Trachurus* *trachurus*) during storage period.^10^^,^^15^^,^^28^^,^^29^

According to the obtained results, lipid hydrolysis and oxidation occurred in trout fillets stored at 4 ˚C during the period of study. Based on present results, although lipid hydrolysis (FFA) was not affected by α-tocopherol acetate solution, successful inhibition of lipid oxidation (PV and TBA) in refrigerated trout fillets was possible with dip treatment in α-tocopherol acetate solutions (200 and 500 mg kg^-1^ flesh). But α-tocopherol acetate solution at lower dose (200 mg kg^-1 ^flesh) showed a higher positive benefit on improving oxidative stability of trout fillets. These results demonstrate that α-tocopherol acetate solution (200 mg kg^-1^ flesh) due to its good antioxidant potential can be used in fish industry. Further studies should be carried out to determine the minimum optimum α-tocopherol acetate level and clarify the antioxidant activity of pre-storage α-tocopherol acetate on seafood quality under different storage conditions. Although lipid oxidation has great potential for evaluating freshness of fish, the eating quality and bacterial load of fish are the important attributes that influence the acceptability of fish to consumers. It seems that to evaluate the freshness and shelf-life of fish, the combination of lipid oxidation changes along with the microbial properties, the organoleptic and nutritional quality of fish should be further investigated. 
